# Major Depression and the Degree of Suicidality: Results of the European Group for the Study of Resistant Depression (GSRD)

**DOI:** 10.1093/ijnp/pyy009

**Published:** 2018-02-23

**Authors:** Markus Dold, Lucie Bartova, Gernot Fugger, Alexander Kautzky, Daniel Souery, Julien Mendlewicz, George N Papadimitriou, Dimitris Dikeos, Panagiotis Ferentinos, Stefano Porcelli, Alessandro Serretti, Joseph Zohar, Stuart Montgomery, Siegfried Kasper

**Affiliations:** 1 Department of Psychiatry and Psychotherapy, Medical University of Vienna, Vienna, Austria; 2 Université Libre de Bruxelles, Brussels, Belgium; 3 Psy Pluriel, Centre Européen de Psychologie Médicale, Brussels, Belgium; 4 First Department of Psychiatry, Medical School, National and Kapodistrian University of Athens, Eginition Hospital, Athens, Greece; 5 Second Department of Psychiatry, Medical School, National and Kapodistrian University of Athens, Attikon General Hospital, Athens, Greece; 6 Department of Biomedical and NeuroMotor Sciences, University of Bologna, Bologna, Italy; 7 Sackler Faculty of Medicine, Tel Aviv University, Tel Aviv, Israel; 8 Imperial College, University of London, London, United Kingdom

**Keywords:** augmentation/combination treatment, major depressive disorder, suicidality, treatment response

## Abstract

**Background:**

This European multicenter study aimed to elucidate suicidality in major depressive disorder. Previous surveys suggest a prevalence of suicidality in major depressive disorder of ≥50%, but little is known about the association of different degrees of suicidality with socio-demographic, psychosocial, and clinical characteristics.

**Methods:**

We stratified 1410 major depressive disorder patients into 3 categories of suicidality based on the Hamilton Rating Scale for Depression item 3 (suicidality) ratings (0=no suicidality; 1–2=mild/moderate suicidality; 3–4=severe suicidality). Chi-squared tests, analyses of covariance, and Spearman correlation analyses were applied for the data analyses.

**Results:**

The prevalence rate of suicidality in major depressive disorder amounted to 46.67% (Hamilton Rating Scale for Depression item 3 score ≥1). 53.33% were allocated into the no, 38.44% into the mild/moderate, and 8.23% into the severe suicidality patient group. Due to the stratification of our major depressive disorder patient sample according to different levels of suicidality, we identified some socio-demographic, psychosocial, and clinical variables differentiating from the patient group without suicidality already in presence of mild/moderate suicidality (depressive symptom severity, treatment resistance, psychotic features, add-on medications in general), whereas others separated only when severe suicidality was manifest (inpatient treatment, augmentation with antipsychotics and benzodiazepines, melancholic features, somatic comorbidities).

**Conclusions:**

As even mild/moderate suicidality is associated with a failure of achieving treatment response, adequate recognition of this condition should be ensured in the clinical practice.

**Significance Statement**

Suicidality represents a meaningful clinical challenge in the treatment of patients suffering from unipolar depression. In our European multicenter study, we examined socio-demographic, psychosocial, and clinical features in different degrees of suicidality (no, mild/moderate, and severe suicidality). For some variables (depressive symptom severity, treatment resistance, psychotic features, add-on medications in general), we found significant differences already between patients exhibiting mild/moderate severity and those without suicidality. For other investigated variables, however, significant differences compared with the absence of suicidality could be determined only in case of the presence of severe suicidality. This applies to the variables inpatient treatment, augmentation with antipsychotics and benzodiazepines, melancholic features, and somatic comorbidities.

## Introduction

Major depressive disorder (MDD) represents one of the most common medical illnesses worldwide with a median 12-month prevalence rate of 6.9% (Wittchen et al., 2011) and a lifetime prevalence rate varying between 11.2% and 16% (Bauer et al., 2013; Dold and Kasper, 2017). In European countries, more than approximately 30 million people are affected by unipolar depressive disorders (Wittchen et al., 2011), which cause diminished quality of life, functional impairment, and considerable economic burden (Bandelow et al., 2008; Wittchen et al., 2011; Bauer et al., 2013). It is estimated that up to 10% of all MDD patients attempt suicide (Holma et al., 2014), and population-based surveys suggest for inpatients with MDD a 20-fold increased risk of completed suicide compared with the general population (Høyer et al., 2000; Osby et al., 2001). The vast majority of subjects attempting suicide exhibited beforehand a manifestation of suicidality that can be regarded as a strong antecedent for later suicide attempts (Kessler et al., 1999; Brown et al., 2000; Sokero et al., 2003; Vuorilehto et al., 2014). However, inversely only a small proportion of the patients displaying suicidality ultimately attempt suicide (Kessler et al., 1999). Depending on the applied definitions, the prevalence of suicidality amounts to more than 55% in patients with predominant MDD (Asnis et al., 1993; Schaffer et al., 2000; Sokero et al., 2003; Zisook et al., 2009). Despite its clinical significance, little is known regarding the various degrees of suicidality, as most of the trials investigating this condition in MDD stratified their participants into patient groups with vs without suicidality in a dichotomous manner. However, to the best of our knowledge we are not aware of surveys itemizing their study sample according to different categories of suicidality as realized in the present European multicenter research project. In this study, we sought (1) to examine the prevalence of different levels of suicidality in a large naturalistic MDD sample (n=1410), (2) to investigate socio-demographic, psychosocial, and clinical features associated with suicidality, and (3) to explore the differences of these variables between the various degrees of suicidality.

## Methods

### Study Design

From 2011 to 2016, all participants were recruited in the context of the European multicenter project “Clinical and Biological Correlates of Resistant Depression and Related Phenotypes (TRD 3)” of the Group for the Study of Resistant Depression (GSRD). Altogether, 10 centers in 8 European countries were involved in this study, which was approved by the ethics committees of all participating centers. Each participant had to provide written informed consent prior to study entry.

### Patients

Study participants were men and women, aged 18 years and older, who met the DSM-IV-TR criteria for MDD. The diagnosis had to be established by the Mini-International Neuropsychiatric Interview (Sheehan et al., 1998). A further inclusion criterion was at least one adequate previous treatment with antidepressant drugs (≥4 weeks in sufficient dose; supplementary Table 1). Patients were excluded from this study if they presented any current primary psychiatric disorder other than MDD, any substance disorder (except nicotine and caffeine) within the previous 6 months, or any severe personality disorder.

### Data Collection

The patients’ socio-demographic, psychosocial, and clinical information were gathered within a detailed clinical interview conducted by specifically trained psychiatrists and specific questionnaires (cross-sectional data collection process). Lifetime and current diagnoses, course of illness, comorbidities, and psychopathological features were evaluated on the basis of the MINI modified for the GSRD. The degree of suicidality was determined by the item 3 (suicidality) of the Hamilton Rating Scale for Depression (HAM-D) (Hamilton, 1960). Severity of depression was assessed using the Montgomery and Åsberg Depression Rating Scale (MADRS) (Montgomery and Asberg, 1979) and the 17- and 21-item HAM-D. Additionally, the symptom severity at the onset of the present MDD episode was evaluated by retrospective MADRS scores. These scores were estimated according to the patient’s statements and information obtained from medical records. Hence, symptom changes during the current depressive episode could be operationalized by calculating MADRS total score changes (retrospective MADRS score - present MADRS score). Treatment nonresponse was defined by a MADRS total cut-off score of ≥22 and <50% MADRS total score improvement after at least one adequate antidepressant trial (≥4 weeks duration in adequate dose; see supplementary Table 1). Treatment resistance was defined as the failure to respond to ≥2 consecutive adequate antidepressant trials.

### Statistical Analyses

All participants were stratified into 3 categories of suicidality based on the HAM-D item 3 (suicidality) ratings: patients with an item-score of 0 (absent) represent the no suicidality study group, participants with item-scores of 1 (feels life is not worth living) or 2 (wishes he were dead or any thoughts of possible death to self) were grouped together in the mild/moderate suicidality group, and patients with item-scores of 3 (suicide ideas or gesture) or 4 (suicide attempts) represent the severe suicidality patient group. Descriptive statistics (means, SD, and/or percentages) were used to present the characteristics of the 3 patient groups. To identify statistical significance of categorical variables between the 3 levels of suicidality, chi-squared tests were performed. ANCOVA with the suicidality groups (fixed effect) and recruitment sites (random factor) as variables were used for analyzing continuous characteristics. Posthoc analyses were accomplished to compare the 3 suicidality groups with each other in pairs. Statistical significance was conservatively set at a *P* value of ≤.0011, corresponding to the Bonferroni correction for multiple comparisons (45 variables). Furthermore, we performed Spearman correlation analyses to examine the association between the HAM-D item 3 subscores and the investigated continuous variables. The data were analyzed using SPSS software, version 24.0.

## Results

### Study Sample

A total of 1410 MDD patients could be included in this study. Socio-demographic, psychosocial, and clinical features of the patient sample are shown in Table 1. Of our participants, 33.12% were male, 96.17% were Caucasians, and the mean age was 50.28±14.11 years. 90.99% exhibited recurrent MDD, 10.92% psychotic features, 60.71% melancholic features, and 2.34% atypical features. 46.31% suffered from at least one somatic comorbidity and the most often identified psychiatric comorbidity was an anxiety disorder (20.85%). 34.61% of the participants were treated in an inpatient setting. The mean MADRS total score was 24.61±11.29 and the 21-item HAM-D amounted to 19.78±9.05 points. Benzodiazepines (BZD)/BZD-like drugs (33.05%), antidepressants (29.50%), and antipsychotics (25.67%) were the most frequently prescribed compounds for augmentation/combination medications.

**Table 1.** Patients’ Demographic, Clinical, and Treatment Characteristics for the Comparison of MDD Patients with No vs Mild/Moderate vs Severe Suicidality

Characteristics	MDD Sample total (n=1410)	No Suicidality group score 0 (n=752)	Mild/Moderate Suicidality group (n=542)	Severe Suicidality group (n=116)	x^2^/F	*P* Value (ANCOVA/x^2^)
Gender, n (%)						
Male	467 (33.12)	236 (31.38)	186 (34.32)	45 (38.79)	3.06	.2165
Female	943 (66.88)	516 (68.62)	356 (65.68)	71 (61.21)		
Age, mean (SD), y	50.28 (14.11)	50.79 (14.39)	48.15 (13.35)	55.46 (14.35)	2.13	.13
Marital status, n (%)						
Married / live with	703 (49.86)	405 (53.86)	247 (45.57)	51 (43.97)	10.40	.0056
Single/divorced/separated/widowed	707 (50.14)	347 (46.14)	295 (54.43)	65 (56.03)		
Ethnic origin, n (%)						
Caucasian	1356 (96.17)	721 (95.88)	520 (95.94)	115 (99.14)	3.03	.2903
Weight, mean (SD), kg	73.23 (16.80)	72.58 (16.69)	73.31 (16.26)	77.09 (19.46)	1.04	.36
Highest level of education and/or degree, n (%) (n=1395)						
University education/non-university high education/high level general education	755 (54.12)	426 (57.41)	281 (52.04)	48 (42.48)	10.35	.0057
General secondary / technical education/ elementary school/ none	640 (45.88)	316 (42.59)	259 (47.96)	65 (57.52)		
Occupational status, n (%) (n=1408)						
Employed	659 (46.80)	379 (50.40)	243 (44.92)	37 (32.17)	14.56	.0007
Without occupation	749 (53.20)	373 (49.60)	298 (55.08)	78 (67.83)		
Depressive episode, n (%)						
Single	127 (9.01)	90 (11.97)	27 (4.98)	10 (8.62)	18.78	<.0001
Recurrent	1283 (90.99)	662 (88.03)	515 (95.02)	106 (91.38)		
With psychotic features	154 (10.92)	51 (6.78)	81 (14.94)	22 (18.97)	29.98	<.0001
With melancholic features	856 (60.71)	440 (58.51)	321 (59.23)	95 (81.90)	23.86	<.0001
With atypical features	33 (2.34)	20 (2.66)	8 (1.48)	5 (4.31)	4.08	.1303
Setting, n (%)						
Inpatient	488 (34.61)	213 (28.32)	194 (35.79)	81 (69.83)	77.04	<.0001
Outpatient	922 (65.39)	539 (71.68)	348 (64.21)	35 (30.17)		
Psychiatric comorbidities, n (%)						
Any anxiety disorder	294 (20.85)	150 (19.95)	122 (22.51)	22 (18.97)	1.53	.4664
Generalized anxiety disorder	151 (10.71)	73 (9.71)	71 (13.10)	7 (6.03)	6.68	.0355
Panic disorder	114 (8.09)	63 (8.38)	40 (7.38)	11 (9.48)	0.75	.6859
Agoraphobia	113 (8.01)	57 (7.58)	46 (8.49)	10 (8.62)	0.42	.8127
Social phobia	45 (3.19)	18 (2.39)	24 (4.43)	3 (2.59)	4.37	.1125
Obsessive-compulsive disorder	22 (1.56)	10 (1.33)	9 (1.66)	3 (2.59)	1.10	.5765
Posttraumatic stress disorder	20 (1.42)	9 (1.20)	8 (1.48)	3 (2.59)	1.40	.4945
Anorexia nervosa	1 (0.07)	0 (0.00)	1 (0.18)	0 (0.00)	1.60	.4488
Bulimia nervosa	8 (0.57)	2 (0.27)	6 (1.11)	0 (0.00)	4.67	.0968
Somatic comorbidities, n (%)						
Any somatic comorbidity	653 (46.31)	341 (45.35)	242 (44.65)	70 (60.34)	10.07	.0065
Hypertension	267 (18.94)	146 (19.41)	88 (16.24)	33 (28.45)	9.52	.0086
Thyroid disease	204 (14.47)	114 (15.16)	70 (12.92)	20 (17.24)	2.07	.3556
Migraine	156 (11.06)	72 (9.57)	71 (13.10)	13 (11.21)	3.98	.1367
Diabetes	84 (5.96)	35 (4.65)	31 (5.72)	18 (15.52)	21.26	<.0001
Heart disease	72 (5.11)	38 (5.05)	19 (3.51)	15 (12.93)	17.53	.0002
Arthritis	65 (4.61)	24 (3.19)	32 (5.90)	9 (7.76)	8.12	.0172
Asthma	48 (3.40)	22 (2.93)	19 (3.51)	7 (6.03)	2.98	.2252
HAM-D total 21-item, mean (SD)	19.78 (9.05)	16.37 (8.71)	22.94 (7.21)	27.08 (9.32)	48.33	<.0001*
*Post-hoc (Bonferoni, LSD, Tukey-HSD): significant differences between all 3 study groups						
HAM-D total 17-item, mean (SD)	18.76 (8.74)	15.46 (8.42)	21.89 (7.06)	25.47 (8.68)	52.99	<.0001*
*Post-hoc (Bonferoni, LSD, Tukey-HSD): significant differences between all 3 study groups						
MADRS total, mean (SD)	24.61 (11.29)	19.66 (10.93)	29.50 (8.01)	33.79 (10.95)	66.75	<.0001*
*Post-hoc (Bonferoni, LSD, Tukey-HSD): significant differences between all 3 study groups						
MADRS total at onset of current MDD episode, mean (SD)	34.06 (7.70)	31.87 (7.36)	35.75 (6.79)	40.36 (8.39)	13.33	<.0001*
*Post-hoc (Bonferoni, LSD, Tukey-HSD): significant differences between all 3 study groups						
MADRS total change (present MADRS - retrospective MADRS), mean (SD)	-9.36 (10.80)	-12.10 (11.75)	-6.17 (8.10)	-6.54 (10.79)	22.08	<.0001*
*Post-hoc (Bonferoni, LSD, Tukey-HSD): significant differences between all 3 study groups with the exception of the comparison mild/moderate vs severe suicidality						
Treatment response, n (%)						
Response	346 (24.54)	287 (38.16)	46 (8.49)	13 (11.21)	173.87	<.0001
Nonresponse	492 (34.89)	243 (32.31)	207 (38.19)	42 (36.21)		
Resistance	572 (40.57)	222 (29.52)	289 (53.32)	61 (52.59)		
Psychopharmacotherapy						
Number of psychiatric drugs, mean (SD)	2.18 (1.22)	2.01 (1.17)	2.27 (1.24)	2.92 (1.13)	9.51	<.0001*
*Post-hoc (Bonferoni, LSD, Tukey-HSD): significant differences between all 3 study groups						
Polypsychopharmacy, n (%)	855 (60.64)	407 (54.12)	345 (63.65)	103 (88.79)	53.97	<.0001
Monotherapy, n (%)	555 (39.36)	345 (45.88)	197 (36.35)	13 (11.21)		
Administered first-line antidepressant (in the current MDD episode), n (%)						
Selective serotonin reuptake inhibitors	734 (52.06)	433 (57.58)	257 (47.42)	44 (37.93)	49.05	.0003
Serotonin-norepinephrine reuptake inhibitors	336 (23.83)	150 (19.95)	147 (27.12)	39 (33.62)		
Noradrenergic and specific serotonergic antidepressants	121 (8.58)	67 (8.91)	40 (7.38)	14 (12.07)		
Tricyclic antidepressants	74 (5.25)	35 (4.65)	29 (5.35)	10 (8.62)		
Agomelatine	69 (4.89)	13 (1.73)	15 (2.77)	4 (3.45)		
Noradrenaline-dopamine reuptake inhibitors	32 (2.27)	32 (4.26)	36 (6.64)	1 (0.86)		
Serotonin antagonist and reuptake inhibitors	28 (1.99)	14 (1.86)	12 (2.21)	2 (1.72)		
Vortioxetine	6 (0.43)	2 (0.27)	1 (0.18)	0 (0.00)		
Monoamine oxidase inhibitors	5 (0.35)	1 (0.13)	2 (0.37)	2 (1.72)		
Noradrenaline reuptake inhibitors	3 (0.21)	3 (0.40)	3 (0.55)	0 (0.00)		
Tianeptine	2 (0.14)	2 (0.27)	0 (0.00)	0 (0.00)		
Fluoxetine equivalents, mean (SD), mg/d	39.86 (20.78)	37.53 (21.17)	42.12 (19.64)	45.06 (21.47)	7.90	.0008*
*Post-hoc (Bonferoni, LSD, Tukey-HSD): significant differences between all 3 study groups with the exception of the comparison mild/moderate vs severe suicidality						
Applied psychopharmacological combination and augmentation strategies (in addition to the ongoing antidepressant treatment), n (%)						
Combination with at least 1 additional antidepressant	416 (29.50)	190 (25.27)	175 (32.29)	51 (43.97)	19.94	<.0001
Augmentation with at least 1 antipsychotic drug	362 (25.67)	161 (21.41)	146 (26.94)	55 (47.41)	34.18	<.0001
Augmentation with at least 1 mood stabilizer	159 (11.28)	74 (9.84)	65 (11.99)	20 (17.24)	5.85	.0536
Augmentation with at least 1 BZD/BZD-like drug	466 (33.05)	208 (27.66)	192 (35.42)	66 (56.90)	38.99	<.0001
Augmentation with at least 1 low-potency antipsychotic^*a*^	91 (6.45)	42 (5.59)	32 (5.90)	17 (14.66)	14.14	.0009
Augmentation with pregabalin	102 (7.23)	48 (6.38)	44 (8.12)	10 (8.62)	1.78	.4116

Abbreviations: BZD,benzodiazepines; HAM-D,Hamilton Rating Scale for Depression; MADRS,Montgomery Åsberg Depression Rating Scale; MDD,major depressive disorder.

The no suicidality group comprised MDD patients with a HAM-D item 3 score of 0 (absent), the mild/moderate suicidality study group incorporated participants with HAM-D item 3 scores of 1 (feels life is not worth living) or 2 (wishes he were dead or any thoughts of possible death to self), and patients with HAM-D item 3 scores of 3 (suicide ideas or gesture) or 4 (suicide attempts) represented the severe suicidality patient group.

The *P* values indicated in bold were significant after Bonferroni correction.

^*a*^Comprising the so-called low-potency first-generation antipsychotics and the SGA quetiapine <100 mg/d.

### Prevalence of Suicidality Itemized by Severity

**Figure 1.**
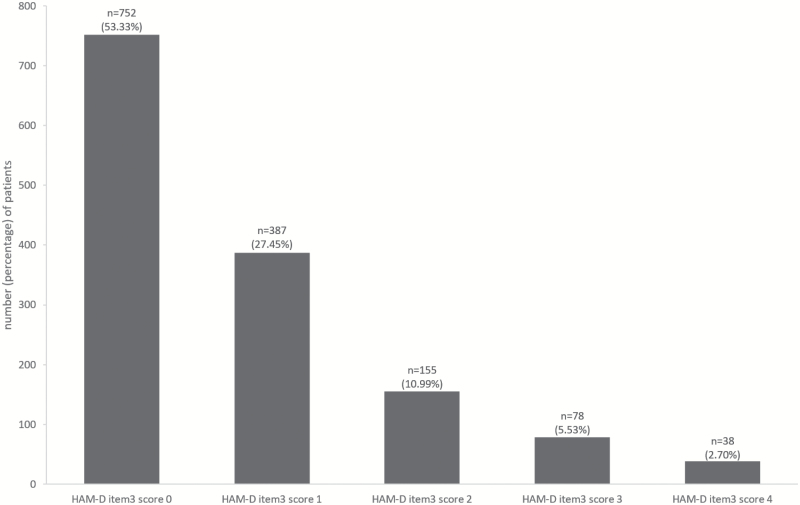
Hamilton Rating Scale for Depression (HAM-D) item 3 (suicidality) ratings.

Overall, 658 of 1410 (46.67%) MDD patients showed suicidality evidenced by a HAM-D item 3 score ≥1. 27.45% met the criteria for HAM-D item 3 score 1, 10.99% for score 2, 5.53% for score 3, and 2.70% for score 4 (Figure 1). Hence, the no suicidality patient group comprised 752 (53.33%) MDD patients, the mild/moderate suicidality group 542 (38.44%) participants, and the severe suicidality group 116 (8.23%) patients.

The numbers and percentages of the patients refer to the whole analyzed study sample comprising 1410 patients with major depressive disorder.

### Socio-Demographic, Psychosocial, and Clinical Variables

Among the investigated socio-demographic, psychosocial, and clinical features, we found the following significant differences between the 3 suicidality groups (severe, mild/moderate, and no suicidality) (Tables 1 and 2; supplementary Tables 2–4). In the patient group with severe suicidality, we determined a higher percentage of patients without occupation (67.83%) compared with the no suicidality group (49.60%, *P*<.0001). Recurrent MDD was more likely in subjects with mild/moderate (95.02%) than without suicidality (88.03%, *P*<.0001). Psychotic symptoms emerged less frequently in the no suicidality group (6.78%) compared with the mild/moderate (14.94%, *P*<.0001) and severe (18.97%, *P*<.0001) suicidality groups. Melancholic features were more often present in MDD patients characterized by severe (81.90%) than mild/moderate (59.23%, *P*<.0001) and no suicidality (58.51%, *P*<.0001). Inpatient treatment was more frequently applied in MDD patients exhibiting severe (69.83%) compared with mild/moderate (35.79%, *P*<.0001) and no suicidality (28.32%, *P*<.0001).

**Table 2.** Overview Regarding the Statistically Significant Between-Group Differences (No vs Mild/Moderate vs Severe Suicidality) and the Accompanying Posthoc Tests (No vs Mild/Moderate Suicidality, No vs Severe Suicidality, Mild/Moderate vs Severe Suicidality)

Characteristics	No vs Mild/Moderate vs Severe Suicidality	No vs Mild/Moderate Suicidality	No vs Severe Suicidality	Mild/Moderate vs Severe Suicidality
Occupational status, n (%)	**X**		**X**	
Single vs recurrent episodes, n (%)	**X**	**X**		
Presence of psychotic features, n (%)	**X**	**X**	**X**	
Presence of melancholic features, n (%)	**X**		**X**	**X**
Inpatient vs outpatient treatment, n (%)	**X**		**X**	**X**
Comorbid diabetes, n (%)	**X**		**X**	**X**
Comorbid heart disease, n (%)	**X**		**X**	**X**
HAM-D total 21-item, mean (SD)	**X**	**X**	**X**	**X**
HAM-D total 17-item, mean (SD)	**X**	**X**	**X**	**X**
MADRS total, mean (SD)	**X**	**X**	**X**	**X**
MADRS total at onset of current MDD episode, mean (SD)	**X**	**X**	**X**	**X**
MADRS total change (present MADRS - retrospective MADRS), mean (SD)	**X**	**X**	**X**	
Treatment response (response vs nonresponse vs resistance), n (%)	**X**	**X**	**X**	
Number of psychiatric drugs, mean (SD)	**X**	**X**	**X**	**X**
Poly- vs monopsychopharmacy, n (%)	**X**	**X**	**X**	**X**
Administered first-line antidepressant (in the current MDD episode), n (%)	**X**		**X**	
Fluoxetine equivalents, mean (SD), mg/d	**X**	**X**	**X**	
Combination with at least 1 additional antidepressant, n (%)	**X**		**X**	
Augmentation with at least 1 antipsychotic drug, n (%)	**X**		**X**	**X**
Augmentation with at least 1 BZD/BZD-like drug, n (%)	**X**		**X**	**X**
Augmentation with at least 1 low-potency antipsychotic^a^, n (%)	**X**		**X**	**X**

Abbreviations: BZD,benzodiazepines; HAM-D,Hamilton Rating Scale for Depression; MADRS,Montgomery Åsberg Depression Rating Scale; MDD,major depressive disorder.

This overview table summarizes the statistically significant differences between the 3 analyzed patient groups (no vs mild/moderate vs severe suicidality) and the accompanying posthoc tests (no vs mild/moderate suicidality, no vs severe suicidality, mild/moderate vs severe suicidality) determined in the chi-squared tests (categorical variables) and ANCOVA (continuous variables). An “X” indicates the presence of a significant between-group difference, whereas an empty square represents the absence of a significant difference for the relevant comparison. Table 1 and Supplementary online tables 2–4 provide the corresponding numerical results of the statistical data analyses.

^*a*^Comprising the so-called low-potency first-generation antipsychotics and the SGA quetiapine <100 mg/d.

### Comorbidities

We found a significantly higher proportion of comorbid diabetes and heart disease in patients with severe suicidality (15.52% and 12.93%, respectively) than mild/moderate (5.72% and 3.51%, respectively) and no suicidality (4.65% and 5.05%, respectively), whereas no significant differences emerged in the comparison of mild/moderate to no suicidality.

### Depressive Symptom Severity and Treatment Response

**Figure 2.**
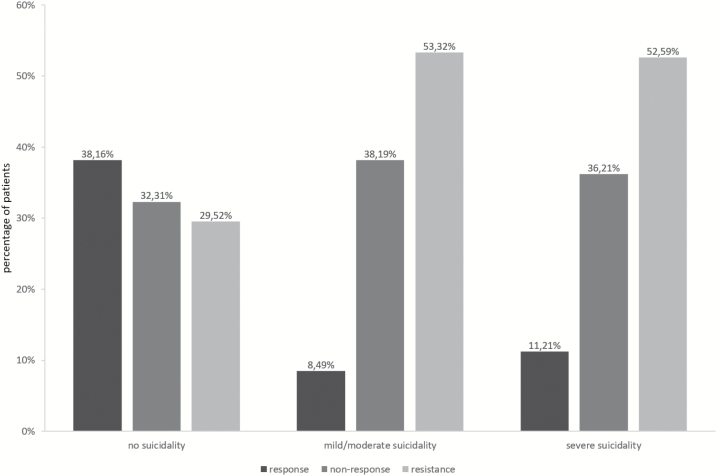
Treatment response, nonresponse, and resistance rates in the no, mild/moderate, and severe suicidality patient groups.

With respect to the depressive symptom severity measured by mean 17-item and 21-item HAM-D scores as well as present and retrospective MADRS scores, we found significantly higher mean scores for patients suffering from severe and mild/moderate suicidality compared with no suicidality and from severe compared with mild/moderate suicidality. When analyzing MADRS total change, we determined a higher reduction in the no suicidality group than in the severe (mean difference: 5.56 points) and mild/moderate (mean difference: 5.93 points) suicidality groups, whereas there was no significant difference between mild/moderate and severe suicidality. Accordingly, the response rates were higher in the no suicidality group (38.16%) compared with the mild/moderate (8.49%, *P*<.0001) and severe suicidality groups (11.21%, *P*<.0001) (Figure 2). Inversely, treatment resistance rates were lower in patients without (29.52%) than with mild/moderate (53.32%, *P*<.0001) and severe suicidality (52.59%, *P*<.0001). No significant differences in response and resistance rates were identified when comparing severe to mild/moderate suicidality.

### Psychopharmacotherapy

Augmentation/combination treatment strategies in general were more frequently established the higher the degree of suicidality was: 88.79% of the patients with severe suicidality received augmentation/combination medications and the mean number of concurrently prescribed psychiatric drugs amounted to 2.92±1.13. For mild/moderate and no suicidality, the polypharmacy rates were 63.65% and 54.12% and the mean numbers of psychiatric drugs were 2.27±1.24 and 2.01±1.17, respectively. We determined statistical significances between all 3 categories of suicidality for both, the polypharmacy rates and the mean number of drugs (*P*<.0001 for all comparisons). With respect to the individual augmentation/combination strategies, we found a significantly higher proportion of patients receiving antidepressant combination treatment, augmentation with antipsychotic drugs, add-on treatment with BZD/BZD-like agents, and adjunctive medication with low-potency antipsychotics (comprising the so-called low-potency first-generation antipsychotics and the SGA quetiapine <100 mg/d) in the severe compared with the no suicidality group. In addition, the differences were significant between severe and mild/moderate suicidality for augmentation treatment with antipsychotics, BZD/BZD-like drugs, and low-potency antipsychotics, but not for antidepressant combination medications. Furthermore, no significant difference for any individual augmentation/combination strategy could be identified for the comparison mild/moderate vs no suicidality.

In terms of the established first-line antidepressant treatment, we could see that selective serotonin reuptake inhibitors (SSRIs) were more likely administered in patients without (57.58%) than with severe suicidality (37.93%, *P*<.0001). Inversely, serotonin-norepinephrine reuptake inhibitors (SNRIs) were more frequently prescribed when severe suicidality was present (33.62%) in comparison to the absence of suicidality (19.95%, *P*<.0001). Moreover, we found higher prescription rates of the noradrenergic and specific serotonergic antidepressants (NaSSAs) and tricyclic antidepressants in the severe compared with the no suicidality group (12.07% vs 8.91% and 8.62% vs 4.65%, respectively, *P*<.0001 for all comparisons). The dosing of the administered antidepressants expressed in fluoxetine equivalents was significantly higher in the severe suicidality group (45.06±21.47 mg/d) than in the mild/moderate (42.12±19.64 mg/d) and no suicidality group (37.53±21.17 mg/d).

### Correlation Analyses

Applying Spearman correlation analyses (Table 3), we found a positive correlation between the HAM-D item 3 subscores and the variables 17-item HAM-D total score (r=.41, *P*<.0001), 21-item HAM-D total score (r=.42, *P*<.0001), current MADRS total score (r=.51, *P*<.0001), retrospective MADRS total score (r=.35, *P*<.0001), MADRS total score change (r=.24, *P*<.0001), mean number of simultaneously dispensed psychiatric drugs (r=.20, *P*<.0001), and antidepressant dosing expressed by fluoxetine equivalents (r=.15, *P*<.0001).

**Table 3.** Spearman Correlation Analyses Investigating the Association between the Hamilton Rating Scale for Depression (HAM-D) Item 3 (Suicidality) Subscores and the Continuous Demographic and Clinical Variables

Characteristics	r	*P* Value
Age, mean (SD), y	.0154	.5648
Weight, mean (SD), kg	.0048	.0736
HAM-D total 17-item, mean (SD)	.4147	<.0001
HAM-D total 21-item, mean (SD)	.4174	<.0001
MADRS total, mean (SD)	.5075	<.0001
MADRS total at onset of current MDD episode, mean (SD)	.3452	<.0001
MADRS total change (present MADRS - retrospective MADRS), mean (SD)	.2413	<.0001
Number of psychiatric drugs, mean (SD)	.1987	<.0001
Fluoxetine equivalents, mean (SD), mg/d	.1451	<.0001

Abbreviations: HAM-D,Hamilton Rating Scale for Depression; MADRS,Montgomery Åsberg Depression Rating Scale; MDD,major depressive disorder.

The *P* values indicated in bold were significant after Bonferroni correction.

## Discussion

A major finding of this European multicenter, cross-sectional study comprising 1410 MDD patients represents the observation that the higher the degree of suicidality was, the higher was the depressive symptom severity measured by various rating scales (current and retrospective MADRS, HAM-D). With regard to treatment response patterns (MADRS change, response status measurement), we found that mild/moderate and severe suicidality differentiated both significantly from the absence of suicidality. Concerning the realization of inpatient treatment, antipsychotic augmentation treatment, add-on medication with BZD/BZD-like drugs, the occurrence of melancholic features, and the presence of comorbid diabetes and heart disease however, we could determine that severe suicidality separated significantly from mild/moderate and no suicidality without identifying significant differences between no and mild/moderate suicidality.

### Prevalence of Suicidality in MDD

A total 46.67% of the 1410 MDD patients participating in our research project exhibited suicidality measured by the HAM-D item 3 (suicidality) score. This observed prevalence rate can be regarded as in agreement with other trial results although some studies revealed slightly higher rates (Asnis et al., 1993; Schaffer et al., 2000; Sokero et al., 2003; Zisook et al., 2009). However, most of these surveys assessed suicidality before implementing adequate antidepressant treatment (Zisook et al., 2009, 2011; Morris et al., 2010), whereas our patient sample already received a course of antidepressants before the cross-sectional data collection process and subsequently comprised also treatment responders. This methodological difference might account to the slightly lower prevalence of suicidality in our investigation. Another meaningful variation from previous trials on this research question represents the allocation to the examined patients groups. While the vast majority of previous studies compared patients with suicidality with those without suicidality in a dichotomous manner, we expanded this approach and stratified our MDD participants according to different degrees of suicidality (no, mild/moderate, and severe suicidality). The study group assignment was based on the item 3 subscore (suicidality) of the HAM-D, which could be shown to be a valid approach to adequately assess suicidality (Desseilles et al., 2012).

The use of the HAM-D item 3 (suicidality) score for the evaluation of suicidality was also applied in a large number of clinical trials and meta-analyses investigating the suicide risk of various antidepressant drugs (Beasley et al., 1991; Letizia et al., 1996; Acharya et al., 2006). However, the criticism of the FDA on the HAM-D item 3 assessment should be considered in this regard, as it led subsequently to the development of a further rating instrument for the appraisal of suicidal ideation and behavior in clinical and research settings, the Columbia–Suicide Severity Rating Scale (Posner et al., 2011).

In our trial, 38.44% of all participants fulfilled the predefined criteria for mild/moderate suicidality (HAM-D item 3 score 1 or 2) and 8.23% for severe suicidality (HAM-D item 3 score ≥3). In this context, it should be taken into account that we allocated already patients with a HAM-D item 3 score 1 to the mild/moderate suicidality group, whereas some other studies (Vuorilehto et al., 2014) defined the cut-off score on 2 or even higher. Using such a high cut-off score would cause an exclusion of patients with mild suicidality from the suicidality study groups, and the relevant patient data were subsequently analyzed in the control group without suicidality. This is why we a priori decided to itemize our MDD patient sample according to different categories of suicidality. Due to this methodological measure, we could uncover that some socio-demographic and clinical variables differentiated from the patient group without suicidality already when mild/moderate suicidality symptoms were present (depressive symptom severity, treatment resistance), whereas others only separated just in cases of severe suicidality (occupational status, inpatient treatment, add-on pharmacotherapy with antipsychotics and BZD/BZD-like drugs, comorbid diabetes and heart disease, melancholic features).

The present study aimed to investigate the characteristics of different degrees of suicidality in MDD based on the HAM-D item 3 subscores. However, the presence of suicidality does not necessarily lead to later suicide attempts. Therefore, it should be critically considered that suicidality cannot be equated with suicide risk in our study, which did not seek to examine suicide attempts even if the subscore 4 of the HAM-D item 3 represents manifest suicide behavior.

### Suicidality and Depressive Symptom Severity

Regarding the depressive symptomatology, we could determine an association between increased suicidality and high amount of depressive symptom severity in both the descriptive statistics and the correlation analyses. These observations are concordant with those of a number of previously conducted studies (Pages et al., 1997; Sokero et al., 2003; Zisook et al., 2011; Vuorilehto et al., 2014). The findings for other variables can be interpreted in a similar way. For instance, we explored an association between severe suicidality and (1) a higher proportion of MDD patients receiving inpatient treatment, (2) a larger use of augmentation/combination treatment strategies, (3) a higher proportion of psychotic and melancholic features, and (4) a higher amount of subjects without occupation. All these variables can be regarded as parameters for severe symptom burden due to MDD. The observed association between suicidality and symptom severity is particularly remarkable against the backdrop that the HAM-D and MADRS total scores, which served as indicators for the depressive symptom severity in our study, comprise already the relevant subscores measuring suicidal ideation and behavior. Hence, our findings suggest that suicidality represents a common and sensitive measure of the severity of depressive conditions.

### Suicidality and Treatment Response Pattern

One meaningful finding of this study represents the observation that already mild/moderate suicidality was associated with treatment nonresponse and resistance. The difference between mild/moderate and high suicidality on the other hand was not significant. As clinical consequence of these results, adequate recognition of even mild/moderate suicidality in MDD should be ensured in the psychiatric routine care as this condition is associated with a failure of achieving treatment response. However, when interpreting our statistical findings of an association between suicidality and failed treatment response, it should be critically considered that it cannot be concluded that the direction of causality goes from suicidality to treatment nonresponse and resistance. It appears, however, more likely that the failure of achieving treatment response leads to suicidality or that, at least, the relationship can be regarded as bidirectional.

The inverse findings, however, could be determined with respect to the presence of comorbid diabetes and heart disease. For these somatic comorbidities, we could evidence significant between-group differences only for severe (vs no and mild/moderate suicidality), but not for mild/moderate suicidality in comparison to the absence of suicidality. Interestingly, we found no significant between-group differences with regard to the psychiatric comorbidities. These results vary from those of another study in which concurrent social phobia and bulimia nervosa could be identified as potential risk factors for suicidality in MDD patients (Morris et al., 2010). Reasons for these different findings may be attributable to differences in the investigated MDD study sample as Morris et al. (2010) analyzed data of exclusively nonpsychotic outpatients prior to the implementation of psychopharmacotherapy.

### Suicidality and Psychopharmacotherapy

Some data suggest that antidepressant drugs may potentially be associated with increased suicide risk and suicidal behavior in some MDD patients, particularly in adolescents and younger adults. However, recent clinical trial findings were inconsistent in this regard (Montgomery et al., 1995; Fergusson et al., 2005; Gunnell et al., 2005; Juurlink et al., 2006; Möller, 2006, 2008; Barbui et al., 2009; Seemüller et al., 2009; Termorshuizen et al., 2015; Baldessarini et al., 2017). Even if our study did not primarily aim to elucidate the impact of antidepressant pharmacotherapy on the levels of suicidality, we could determine in a naturalistic way that the presence of suicidality led to a shift from SSRIs prescriptions to an increased administration of SNRIs. While 58% of the MDD patients without suicidality received SSRIs as first-line antidepressants, just 20% were treated with SNRIs. The higher the degree of suicidality was, the more narrow the prescription rates of both substance classes (38% for SSRIs and 34% for SNRIs in severe suicidality). The observed higher prescription rates of NaSSAs and tricyclic antidepressants in the severe suicidality patient group correspond to meta-analytic findings suggesting efficacy of frequently used agents of each substance class, mirtazapine and amitriptyline, in subsamples representing severely depressed patients (Kasper et al., 1997). Moreover, the NaSSA mirtazapine was associated with significantly lower suicide risk in a pooled data analysis of 15 placebo-controlled RCTs (Kasper et al., 2010).

Beyond this influence of the different categories of suicidality on the prescription practice of the first-line antidepressants, we could see an association between the levels of suicidality and the administration of augmentation/combination strategies when analyzing the mean number of simultaneously prescribed psychiatric drugs (ANCOVA, correlation analysis). Moreover, the dichotomous rates of patients receiving augmentation/combination treatment rose from 54% (absence of suicidality) to 89% in case of severe suicidality, whereas inversely the number of monotherapy-treated patients decreased from 46% (no suicidality) to 11% (severe suicidality). With respect to the individual dispensed augmentation/combination strategies, add-on treatment with antidepressants, antipsychotics, benzodiazepines, and low-potency antipsychotic drugs were significantly associated with the presence of severe suicidality. It should be mentioned in this regard that growing evidence suggests potent antisuicidal properties of the N-methyl-D-aspartate receptor antagonist ketamine, which is increasingly used to manage acute suicidal crises in MDD (Niciu et al., 2014; Xu et al., 2016; Kraus et al., 2017). However, there is a need for further clinical studies to substantiate its effectiveness in everyday clinical practice.

### Study Limitations

The naturalistic cross-sectional design of our study should be taken into account as potential limitation. With this approach, we aimed to recruit a real-world MDD patient sample. In contrast, patients exhibiting HAM-D item 3 scores of 3 or 4 are commonly excluded from participation in randomized controlled clinical trials due to ethical reasons. Therefore, clinical studies usually examine a MDD population with low degrees of suicidality. On the contrary, the present naturalistic survey did not exclude those patients to improve the generalizability of the study findings. However, our participants were enrolled exclusively from tertiary care settings (European university/academic psychiatric treatment centers). Therefore, our study sample could be not completely representative. Furthermore, we cannot definitely rule out that in some participants, the enrollment process might be biased by the requirement of ≥4-week antidepressant drug treatment as precondition for study entry. With regard to the MADRS score estimation, a potential bias due to a lack of the calculation of inter-rater reliability should be considered. However, all specialists participating in the data collection process received special training in performing the MADRS ratings. A further limitation concerning the data analyses represents a lack of information in terms of previous suicide attempts. Moreover, possible cross-site differences should be taken into account although we considered this issue within the statistical analyses (center was random factor in the ANCOVA).

## Statement of Interest

Dr. Dold has received a travel grant from Janssen-Cilag. Dr. Souery has received grant/research support from GlaxoSmithKline and Lundbeck and he has served as a consultant or on advisory boards for AstraZeneca, Bristol-Myers Squibb, Eli Lilly, Janssen, and Lundbeck. Dr. Mendlewicz is a member of the faculty of the Lundbeck International Neuroscience Foundation and of the advisory board of Servier. Dr. Dikeos has served on speakers' or advisory boards or has received consultancy fees from Angelini, AstraZeneca, Boehringer, Bristol-Myers Squibb, Eli Lilly, Genesis Pharma, GlaxoSmithKline, Janssen, Lundbeck, Organon, Sanofi, Servier, UniPharma, and Wyeth; and he has received unrestricted grants from AstraZeneca and Eli Lilly as director of the Sleep Research Unit of the Eginition Hospital (Athens University). Dr. Ferentinos has received within the last 3 years honoraria from Servier and travel grants from Eli Lilly, Janssen, and Servier. Dr. Serretti is or has been consultant/speaker for Abbott, Abbvie, Angelini, AstraZeneca, Clinical Data, Boheringer, Bristol-Myers Squibb, Eli Lilly, GlaxoSmithKline, Innovapharma, Italfarmaco, Janssen, Lundbeck, Naurex, Pfizer, Polifarma, Sanofi, and Servier. Dr. Zohar has received grant/research support from Lundbeck, Servier, and Pfizer; he has served as a consultant or on advisory boards for Servier, Pfizer, Solvay, and Actelion; and he has served on speakers’ bureaus for Lundbeck, GSK, Jazz, and Solvay. Dr. Montgomery has been a consultant or served on advisory boards for AstraZeneca, Bionevia, Bristol-Myers Squibb, Forest, GlaxoSmithKline, Grunenthal, Intellect Pharma, Johnson & Johnson, Lilly, Lundbeck, Merck, Merz, M’s Science, Neurim, Otsuka, Pierre Fabre, Pfizer, Pharmaneuroboost, Richter, Roche, Sanofi, Sepracor, Servier, Shire, Synosis, Takeda, Theracos, Targacept, Transcept, UBC, Xytis, and Wyeth. Dr. Kasper received grants/research support, consulting fees, and/or honoraria within the last 3 years from Angelini, AOP Orphan Pharmaceuticals AG, AstraZeneca, Eli Lilly, Janssen, KRKA-Pharma, Lundbeck, Neuraxpharm, Pfizer, Pierre Fabre, Schwabe, and Servier. All other authors declare no conflicts of interest.

## Supplementary Material

Supplementary Tables
