# Supplementary material for: Major Depression and the Degree of Suicidality: Results of the European Group for the Study of Resistant Depression (GSRD)
Source: Int J Neuropsychopharmacol. 2018 Feb 23;21(6):539–49. doi: 10.1093/ijnp/pyy009 (PMC6007240; doi:10.1093/ijnp/pyy009)
Supplement: Supplementary Tables [file pyy009_suppl_supplementary_tables.docx]

**Supplementary Table 1. Required Minimum Doses for the Antidepressant Drug Treatment before Study Entry (Administered for ≥4 Weeks)**

| **Antidepressants** | **Required Daily Minimum Dose** |
| --- | --- |
| **Selective serotonin reuptake inhibitors (SSRIs)** | |
| Citalopram | 20 mg |
| Escitalopram | 10 mg |
| Fluoxetine | 20 mg |
| Fluvoxamine | 50 mg |
| Paroxetine | 20 mg |
| Sertraline | 50 mg |
|  |  |
| **Serotonin-noradrenaline reuptake inhibitors (SNRIs)** | |
| Duloxetine | 60 mg |
| Milnacipran | 100 mg |
| Venlafaxine | 75 mg |
|  |  |
| **Tricyclic antidepressants (TCAs)** | |
| Amitryptiline | 150 mg |
| Clomipramine | 150 mg |
| Desimipramine | 150 mg |
| Dosulepine | 200 mg |
| Dothiepin | 200 mg |
| Imipramine | 150 mg |
| Maprotiline | 150 mg |
| Nortriptiline | 100 mg |
| Protriptyline | 75 mg |
|  |  |
| Monoamine oxidase inhibitors (**MAO-I)** | |
| Moclobemide | 450 mg |
| Phenelzine | 60 mg |
|  |  |
| **Other antidepressants** | |
| Agomelatine | 25 mg |
| Amoxapine | 300 mg |
| Bupropion | 300 mg |
| Mianserine | 30 mg |
| Mirtazapine | 15 mg |
| Reboxetine | 8 mg |
| Tianeptine | 37.5 mg |
| Trazodone | 150 mg |

**Supplementary Table 2. Posthoc Comparison of the Analyzed Dichotomous Variables between MDD Patients Exhibiting no vs Mild/Moderate Suicidality**

| **Characteristics** | **MDD Sample Total (n = 1410)** | **No Suicidality Group (n = 752)** | **Mild/Moderate Suicidality Group (n = 542)** | **x^2^/F** | ***P*-Value (ANCOVA/x^2^)** |
| --- | --- | --- | --- | --- | --- |
| Occupational status, n (%) (n = 1407) | | | | | |
| Employed | 659 (46.80) | 379 (50.40) | 243 (44.92) | 3.79 | .0516 |
| Without occupation | 749 (53.20) | 373 (49.60) | 298 (55.08) |  |  |
| Depressive episode, n (%) | | | | | |
| Single | 127 (9.01) | 90 (11.97) | 27 (4.98) | 18.69 | **<.0001** |
| Recurrent | 1283 (90.99) | 662 (88.03) | 515 (95.02) |  |  |
| With psychotic features | 154 (10.92) | 51 (6.78) | 81 (14.94) | 22.91 | **<.0001** |
| With melancholic features | 856 (60.71) | 440 (58.51) | 321 (59.23) | 0.07 | .7967 |
| Setting, n (%) | | | | | |
| Inpatient | 488 (34.61) | 213 (28.32) | 194 (35.79) | 8.15 | .0043 |
| Outpatient | 922 (65.39) | 539 (71.68) | 348 (64.21) |  |  |
| Somatic comorbidities, n (%) | | | | | |
| Diabetes | 84 (5.96) | 35 (4.65) | 31 (5.72) | 0.74 | .3901 |
| Heart disease | 72 (5.11) | 38 (5.05) | 19 (3.51) | 1.79 | .1807 |
| Treatment response (dichotomous), n (%) | | | | | |
| Response (n = 1409) | 346 (24.54) | 287 (38.16) | 46 (8.49) | 156.11 | **<.0001** |
| Non-Response (n = 1409) | 492 (34.89) | 243 (32.31) | 207 (38.19) |  |  |
| Resistance (n = 1409) | 572 (40.57) | 222 (29.52) | 289 (53.32) |  |  |
| Psychopharmacotherapy | | | | | |
| Polypsychopharmacy, n (%) | 855 (60.64) | 407 (54.12) | 345 (63.65) | 11.75 | **<.0001** |
| Monotherapy, n (%) | 555 (39.36) | 345 (45.88) | 197 (36.35) |  |  |
| Administered first-line antidepressant (in the current MDD episode), n (%) | | | | | |
| Selective serotonin reuptake inhibitors | 734 (52.06) | 433 (57.58) | 257 (47.42) | 22.00 | .0151 |
| Serotonin-norepinephrine reuptake inhibitors | 336 (23.83) | 150 (19.95) | 147 (27.12) |  |  |
| Noradrenergic and specific serotonergic antidepressants | 121 (8.58) | 67 (8.91) | 40 (7.38) |  |  |
| Tricyclic antidepressants | 74 (5.25) | 35 (4.65) | 29 (5.35) |  |  |
| Agomelatine | 69 (4.89) | 13 (1.73) | 15 (2.77) |  |  |
| Noradrenaline-dopamine reuptake inhibitors | 32 (2.27) | 32 (4.26) | 36 (6.64) |  |  |
| Serotonin antagonist and reuptake inhibitors | 28 (1.99) | 14 (1.86) | 12 (2.21) |  |  |
| Vortioxetine | 6 (0.43) | 2 (0.27) | 1 (0.18) |  |  |
| Monoamine oxidase inhibitors | 5 (0.35) | 1 (0.13) | 2 (0.37) |  |  |
| Noradrenaline reuptake inhibitors | 3 (0.21) | 3 (0.40) | 3 (0.55) |  |  |
| Tianeptine | 2 (0.14) | 2 (0.27) | 0 (0.00) |  |  |
| Applied psychopharmacological combination and augmentation strategies (in addition to the ongoing antidepressant treatment), n (%) | | | | | |
| Combination with at least 1 additional antidepressant | 415 (29.43) | 190 (25.27) | 174 (32.10) | 7.28 | .0070 |
| Augmentation with at least 1 antipsychotic drug | 361 (25.60) | 161 (21.41) | 146 (26.94) | 5.32 | .0211 |
| Augmentation with at least 1 BZD/BZD-like drug | 465 (32.98) | 208 (27.66) | 192 (35.42) | 8.89 | .0028 |
| Augmentation with at least 1 low-potency antipsychotic*^a^* | 91 (6.45) | 42 (5.59) | 32 (5.90) | 0.06 | .8073 |

Abbreviations: BZD, benzodiazepines; HAM-D, Hamilton Rating Scale for Depression; MADRS, Montgomery Åsberg Depression Rating Scale; MDD = major depressive disorder.

The no suicidality group comprised MDD patients with a HAM-D item 3 score of 0 (absent) and the mild/moderate suicidality patients with HAM-D item 3 scores of 1 (feels life is not worth living) or 2 (wishes he were dead or any thoughts of possible death to self).

The *P* values indicated in bold were significant after Bonferroni correction.

*^a^* Comprising the so-called low-potency first-generation antipsychotics and the SGA quetiapine <100 mg/d.

**Supplementary Table 3. Posthoc Comparison of the Analyzed Dichotomous Variables between MDD Patients with No vs Severe Suicidality**

| **Characteristics** | **MDD Sample Total (n = 1410)** | **No Suicidality Group (n = 752)** | **Severe Suicidality Group (n = 116)** | **x^2^/F** | ***P* Value (ANCOVA/x^2^)** |
| --- | --- | --- | --- | --- | --- |
| Occupational status, n (%) (n = 1407) | | | | | |
| Employed | 659 (46.80) | 379 (50.40) | 37 (32.17) | 13.27 | **<.0001** |
| Without occupation | 749 (53.20) | 373 (49.60) | 78 (67.83) |  |  |
| Depressive episode, n (%) | | | | | |
| Single | 127 (9.01) | 90 (11.97) | 10 (8.62) | 1.11 | .2932 |
| Recurrent | 1283 (90.99) | 662 (88.03) | 106 (91.38) |  |  |
| With psychotic features | 154 (10.92) | 51 (6.78) | 22 (18.97) | 19.37 | **<.0001** |
| With melancholic features | 856 (60.71) | 440 (58.51) | 95 (81.90) | 23.24 | **<.0001** |
| Setting, n (%) | | | | | |
| Inpatient | 488 (34.61) | 213 (28.32) | 81 (69.83) | 77.29 | **<.0001** |
| Outpatient | 922 (65.39) | 539 (71.68) | 35 (30.17) |  |  |
| Somatic comorbidities, n (%) | | | | | |
| Diabetes | 84 (5.96) | 35 (4.65) | 18 (15.52) | 20.69 | **<.0001** |
| Heart disease | 72 (5.11) | 38 (5.05) | 15 (12.93) | 10.88 | **.0010** |
| Treatment response (dichotomous), n (%) | | | | | |
| Response (n = 1409) | 346 (24.54) | 287 (38.16) | 13 (11.21) | 37.99 | **<.0001** |
| Nonresponse (n = 1409) | 492 (34.89) | 243 (32.31) | 42 (36.21) |  |  |
| Resistance (n = 1409) | 572 (40.57) | 222 (29.52) | 61 (52.59) |  |  |
| Psychopharmacotherapy | | | | | |
| Polypsychopharmacy, n (%) | 855 (60.64) | 407 (54.12) | 103 (88.79) | 49.85 | **<.0001** |
| Monotherapy, n (%) | 555 (39.36) | 345 (45.88) | 13 (11.21) |  |  |
| Administered first-line antidepressant (in the current MDD episode), n (%) | | | | | |
| Selective serotonin reuptake inhibitors | 734 (52.06) | 433 (57.58) | 44 (37.93) | 32.83 | **<.0001** |
| Serotonin-norepinephrine reuptake inhibitors | 336 (23.83) | 150 (19.95) | 39 (33.62) |  |  |
| Noradrenergic and specific serotonergic antidepressants | 121 (8.58) | 67 (8.91) | 14 (12.07) |  |  |
| Tricyclic antidepressants | 74 (5.25) | 35 (4.65) | 10 (8.62) |  |  |
| Agomelatine | 69 (4.89) | 13 (1.73) | 4 (3.45) |  |  |
| Noradrenaline-dopamine reuptake inhibitors | 32 (2.27) | 32 (4.26) | 1 (0.86) |  |  |
| Serotonin antagonist and reuptake inhibitors | 28 (1.99) | 14 (1.86) | 2 (1.72) |  |  |
| Vortioxetine | 6 (0.43) | 2 (0.27) | 0 (0.00) |  |  |
| Monoamine oxidase inhibitors | 5 (0.35) | 1 (0.13) | 2 (1.72) |  |  |
| Noradrenaline reuptake inhibitors | 3 (0.21) | 3 (0.40) | 0 (0.00) |  |  |
| Tianeptine | 2 (0.14) | 2 (0.27) | 0 (0.00) |  |  |
| Applied psychopharmacological combination and augmentation strategies (in addition to the ongoing antidepressant treatment), n (%) | | | | | |
| Combination with at least 1 additional antidepressant | 415 (29.43) | 190 (25.27) | 51 (43.97) | 17.52 | **<.0001** |
| Augmentation with at least 1 antipsychotic drug | 361 (25.60) | 161 (21.41) | 54 (46.55) | 34.09 | **<.0001** |
| Augmentation with at least 1 BZD/BZD-like drug | 465 (32.98) | 208 (27.66) | 65 (56.03) | 37.53 | **<.0001** |
| Augmentation with at least 1 low-potency antipsychotic*^a^* | 91 (6.45) | 42 (5.59) | 17 (14.66) | 13.05 | **<.0001** |

Abbreviations: BZD, benzodiazepines; HAM-D, Hamilton Rating Scale for Depression; MADRS, Montgomery Åsberg Depression Rating Scale; MDD = major depressive disorder.

The no suicidality group comprised MDD patients with a HAM-D item 3 score of 0 (absent) and the mild/moderate suicidality patients with HAM-D item 3 scores of 1 (feels life is not worth living) or 2 (wishes he were dead or any thoughts of possible death to self).

The *P* values indicated in bold were significant after Bonferroni correction.

*^a^*Comprising the so-called low-potency first-generation antipsychotics and the SGA quetiapine <100 mg/d.

**Supplementary Table 4. Posthoc Comparison of the Analyzed Dichotomous Variables between MDD Patients Displaying Mild/Moderate vs Severe Suicidality**

| **Characteristics** | **MDD Sample Total (n = 1410)** | **Mild/Moderate Suicidality Group (n = 542)** | **Severe Suicidality Group (n = 116)** | **x^2^/F** | ***P* Value (ANCOVA/x^2^)** |
| --- | --- | --- | --- | --- | --- |
| Occupational status, n (%) (n = 1407) | | | | | |
| Employed | 659 (46.80) | 243 (44.92) | 37 (32.17) | 6.30 | .0121 |
| Without occupation | 749 (53.20) | 298 (55.08) | 78 (67.83) |  |  |
| Depressive episode, n (%) | | | | | |
| Single | 127 (9.01) | 27 (4.98) | 10 (8.62) | 2.38 | .1225 |
| Recurrent | 1283 (90.99) | 515 (95.02) | 106 (91.38) |  |  |
| With psychotic features | 154 (10.92) | 81 (14.94) | 22 (18.97) | 1.17 | .2794 |
| With melancholic features | 856 (60.71) | 321 (59.23) | 95 (81.90) | 21.12 | **<.0001** |
| Setting, n (%) | | | | | |
| Inpatient | 488 (34.61) | 194 (35.79) | 81 (69.83) | 45.50 | **<.0001** |
| Outpatient | 922 (65.39) | 348 (64.21) | 35 (30.17) |  |  |
| Somatic comorbidities, n (%) | | | | | |
| Diabetes | 84 (5.96) | 31 (5.72) | 18 (15.52) | 13.31 | **<.0001** |
| Heart disease | 72 (5.11) | 19 (3.51) | 15 (12.93) | 17.32 | **<.0001** |
| Treatment response (dichotomous), n (%) | | | | | |
| Response (n = 1409) | 346 (24.54) | 46 (8.49) | 13 (11.21) | 0.90 | .6384 |
| Nonresponse (n = 1409) | 492 (34.89) | 207 (38.19) | 42 (36.21) |  |  |
| Resistance (n = 1409) | 572 (40.57) | 289 (53.32) | 61 (52.59) |  |  |
| Psychopharmacotherapy | | | | | |
| Polypsychopharmacy, n (%) | 855 (60.64) | 345 (63.65) | 103 (88.79) | 27.79 | **<.0001** |
| Monotherapy, n (%) | 555 (39.36) | 197 (36.35) | 13 (11.21) |  |  |
| Administered first-line antidepressant (in the current MDD episode), n (%) | | | | | |
| Selective serotonin reuptake inhibitors | 734 (52.06) | 257 (47.42) | 44 (37.93) | 17.27 | .0446 |
| Serotonin-norepinephrine reuptake inhibitors | 336 (23.83) | 147 (27.12) | 39 (33.62) |  |  |
| Noradrenergic and specific serotonergic antidepressants | 121 (8.58) | 40 (7.38) | 14 (12.07) |  |  |
| Tricyclic antidepressants | 74 (5.25) | 29 (5.35) | 10 (8.62) |  |  |
| Agomelatine | 69 (4.89) | 15 (2.77) | 4 (3.45) |  |  |
| Noradrenaline-dopamine reuptake inhibitors | 32 (2.27) | 36 (6.64) | 1 (0.86) |  |  |
| Serotonin antagonist and reuptake inhibitors | 28 (1.99) | 12 (2.21) | 2 (1.72) |  |  |
| Vortioxetine | 6 (0.43) | 1 (0.18) | 0 (0.00) |  |  |
| Monoamine oxidase inhibitors | 5 (0.35) | 2 (0.37) | 2 (1.72) |  |  |
| Noradrenaline reuptake inhibitors | 3 (0.21) | 3 (0.55) | 0 (0.00) |  |  |
| Tianeptine | 2 (0.14) | 0 (0.00) | 0 (0.00) |  |  |
| Applied psychopharmacological combination and augmentation strategies (in addition to the ongoing antidepressant treatment), n (%) | | | | | |
| Combination with at least 1 additional antidepressant | 415 (29.43) | 174 (32.10) | 51 (43.97) | 5.98 | .0145 |
| Augmentation with at least 1 antipsychotic drug | 361 (25.60) | 146 (26.94) | 54 (46.55) | 17.38 | **<.0001** |
| Augmentation with at least 1 BZD/BZD-like drug | 465 (32.98) | 192 (35.42) | 65 (56.03) | 17.05 | **<.0001** |
| Augmentation with at least 1 low-potency antipsychotic*^a^* | 91 (6.45) | 32 (5.90) | 17 (14.66) | 10.62 | **.0011** |

Abbreviations: BZD, benzodiazepines; HAM-D, Hamilton Rating Scale for Depression; MADRS, Montgomery Åsberg Depression Rating Scale; MDD = major depressive disorder.

The no suicidality group comprised MDD patients with a HAM-D item 3 score of 0 (absent) and the mild/moderate suicidality patients with HAM-D item 3 scores of 1 (feels life is not worth living) or 2 (wishes he were dead or any thoughts of possible death to self).

The *P* values indicated in bold were significant after Bonferroni correction.

*^a^*Comprising the so-called low-potency first-generation antipsychotics and the SGA quetiapine <100 mg/d.
